# Substrate Protein Interactions and Methylglyoxal Modifications Reduce the Aggregation Propensity of Human Alpha-A-Crystallin G98R Mutant

**DOI:** 10.3389/fmolb.2022.875205

**Published:** 2022-04-06

**Authors:** Puttur Santhoshkumar, Krishna K. Sharma

**Affiliations:** ^1^ Department of Ophthalmology, University of Missouri, Columbia, MO, United States; ^2^ Department of Biochemistry, University of Missouri, Columbia, MO, United States

**Keywords:** alpha-A-crystallin, G98R mutation, methylglyoxal, chaperone activity, scattering, cataract, stability

## Abstract

The G98R mutation in αA-crystallin is associated with presenile cataract development in humans. Previous studies have indicated that mutant proteins altered structure, decreased stability, increased oligomeric size, loss of chaperone-like activity, and susceptibility to proteolysis could be contributing factors to cataract formation. To evaluate the effect of substrate protein interactions with the mutant protein on cataract formation, we have performed chaperone assays with alcohol dehydrogenase (ADH), citrate synthase (CS), and βB_2_-crystallin (βB_2_), and analyzed the reaction mixtures by multi-angle light scattering (MALS) analysis. It appears that αAG98R protein initially gets stabilized upon interaction with substrate proteins. Analysis of the chaperone-client protein complexes revealed that wild-type αA-crystallin interacts with substrate proteins to form compact complexes leading to a slight increase in oligomeric mass, whereas αAG98R forms less compact and high molecular weight complexes with the substrate, and the resulting complexes continue to increase in size over time. As a result, the soluble complexes formed initially by the mutant protein begin to scatter light and precipitate. We found that the stability and chaperone activity of the αAG98R can be improved by modifying the protein with low concentrations (50 µM) of methylglyoxal (MGO). Incubation of αAG98R protein (1 mg/ml) under aseptic conditions for 30 days at 37°C resulted in precipitation of the mutant protein. In contrast, mutant protein incubations carried out with 50 µM MGO remained soluble and transparent. SDS-PAGE analysis showed gradual autolysis of the mutant protein in the absence of MGO. The average molar mass of the mutant protein oligomers changed from 7,258 ± 12 kDa to 3,950 ± 08 kDa within 60 min of incubation with MGO. There was no further significant change in the molar mass of mutant protein when tested on day 7 of MGO treatment. Our data suggest that the initial stabilization of αAG98R by substrate proteins could delay congenital cataracts’ appearance, and the uncontrolled long-term interaction amongst mutant subunits and substrate proteins could be the rationale behind presenile cataracts formation. The results also demonstrate the potential benefit of low concentrations of MGO in stabilizing mutant chaperone protein(s).

## 1 Introduction

α-Crystallin, one of the major structural proteins of the vertebrate eye lens, is composed of two subunits designated as αA- and αB-crystallins, each having a molecular mass of about 20 kDa (Bloemendal et al., 2004). The subunits in α-crystallin organize to form polydisperse homo or hetero-oligomers *in vitro* that vary in size and assembly (Haley et al., 1998; Narberhaus, 2002; Aquilina et al., 2003; Sprague-Piercy et al., 2021). Besides its structural role, α-crystallins have been shown to suppress the precipitation of unfolded and denaturing proteins (chaperone activity), signifying its importance in maintaining lens transparency (Horwitz, 1992; Kumar and Reddy, 2009). In support of this view, many studies have shown that a loss of chaperone activity is associated with cataractous lenses (Kelley et al., 1993; Derham and Harding, 1997; Sharma and Santhoshkumar, 2009). Unlike classical heat shock proteins, the substrate proteins remain associated with α-crystallins to form high-molecular-weight complexes that grow larger with time and eventually become water-insoluble. Post-translational modifications and chemical insults accelerate the formation of high molecular weight complexes by α-crystallin (Cobb and Petrash, 2002; Sharma and Santhoshkumar, 2009). The α-crystallin-substrate complexes formed during chaperone action *in vitro* resemble high molecular weight complexes formed by α-crystallin in aging human lenses (Lee et al., 1998). Formation of such high molecular weight aggregates *in vivo* results in decreased light transmittance across the lens.

Several missense mutations in the αA-crystallin gene are linked to cataract development in humans (Panda et al., 2016; Sprague-Piercy et al., 2021). In most of these mutants, there is either a loss or gain of arginine residue suggesting the importance of “net charge” in α-crystallin in maintaining the functional integrity of the lens (Biswas et al., 2006). The hereditary mutations caused by the loss of arginine residue in αA-crystallin result in congenital cataract development (Panda et al., 2016; Song et al., 2018). In contrast, the gain of an arginine residue, such as in αAG98R carriers, leads to presenile cataract development (Santhiya et al., 2006). Earlier studies have suggested that αAG98R mutation affects the protein structure, leading to decreased chaperone-like activity resulting in protein aggregation and lens opacification (Singh et al., 2006). Our lab showed that the G98R mutant protein strongly binds to the substrate proteins and exhibits substrate-dependent chaperone activity (Murugesan et al., 2007). We also showed that αAG98R mutant oligomers are unstable at physiological temperatures and dissociate into monomers at protein concentrations below 0.1 mg/ml (Raju et al., 2011). It has been shown that the structure and chaperone function of αAG98R can be improved after interaction with αA-crystallin-derived mini chaperones (Raju et al., 2012), compensating for the mutant’s lost surface charge by increasing the zeta (ζ) potential (Phadte et al., 2018). Chemical chaperone trimethylamine N-oxide was shown to reduce the mutant’s aggregation in cells (Gong et al., 2009). Recently we showed that the instability of G98R protein could be rescued by introducing a site-specific compensatory mutation in the αAG98R crystallin (Phadte et al., 2019). In this study, we have compared the interactions of αA-WT and αAG98R mutant with the substrate proteins to evaluate their effects on αAG98R oligomer stability.

We have also looked at the effects of methylglyoxal (MGO) modification on the structure, stability, and chaperone-like activity of the mutant protein and its contribution to cataract development. MGO, a metabolic dicarbonyl compound, is a major contributor to advanced glycation end-product formation in the lens (Thornalley, 1993; Haik et al., 1994; Shamsi et al., 1998). As the concentration of MGO increases in the lens, it is expected to bind and modify the proteins, eventually leading to protein crosslinking and precipitation (Riley and Harding, 1995; Kumar et al., 2004; Kumar et al., 2007). Incubations of proteins with MGO have shown arginine as the primary target in these modifications, and lysine, histidine, and cysteine are other amino acids that get modified (Derham and Harding, 2002). Hydroimidazolone and argpyrimidine are two adducts that form *in vivo* when MGO reacts with arginine residues in αA-crystallin (Nagaraj et al., 2003; Gangadhariah et al., 2010; Nagaraj et al., 2012). The glycation by MGO abolishes the charge on arginine residue (Biswas et al., 2008). Previous studies have shown that α-crystallin modified by low concentrations (micromolar) of MGO improves its chaperone function (Nagaraj et al., 2003; Nagaraj et al., 2008). A few other studies that tested higher MGO concentrations (millimolar) reported a loss in chaperone activity along with α-crystallin crosslinking (Derham and Harding, 2002; Kumar et al., 2004; Kumar et al., 2007). We hypothesized that transient exposure of αAG98R to low concentrations of MGO would improve the mutant protein’s stability and chaperone activity.

Our study provides a rationale behind the presenile cataract development in αAG98R mutant. We show that the aggregation propensity of αAG98R mutant is transiently alleviated upon interaction with substrate proteins. However, the complexes precipitate earlier than those formed between the WT and substrate proteins. Modification of αAG98R by MGO in the short term improves the stability of the mutant protein, which might also explain the absence of cataracts at birth in individuals with αAG98R mutation.

## 2 Experimental Procedures

### 2.1 Preparation of αAG98R and αAWT-Crystallin

Human αA-crystallin cDNA cloned into pET-23d (+) vector (Novagen, Madison, WI, United States) was used as a template to generate G98R mutation (Murugesan et al., 2007). The mutant and wild-type proteins were expressed in *E. coli* BL21(DE3)pLysS cells (Invitrogen, Carlsbad, CA, United States) and purified as described earlier with minor changes (Santhoshkumar and Sharma, 2006). Briefly, bacterial cell pellet obtained from one-liter culture was suspended in 5 ml lysis buffer containing 50 mM Tris-HCl (pH 8.0), 0.1 M NaCl, and 2 mM EDTA and treated with 50 μl of protease inhibitor cocktail III (Calbiochem, San Diego, CA, United States), 1 mg lysozyme (Worthington Biochemical Corp. Lakewood, NJ, United States) and 10 mM DTT. The cell suspension was treated with 1 μl of benzonase (Sigma, St. Louis, MO, United States) and incubated at 37°C on a shaking platform for 30 min. The extract was centrifuged at 17,000 g for 1 h. The αAG98R protein partitioned into insoluble fractions was washed and dissolved in Tris-HCl (pH 7.2) buffer containing 1 mM EDTA and 6M urea. The urea dissolved supernatant was filtered and loaded into a Q-Sepharose Fast Flow ion-exchange column equilibrated with Tris-EDTA buffer. The protein was eluted using a stepwise gradient of 1 M NaCl in 50 mM Tris-HCl (pH 7.2) containing 1 mM EDTA at 1 ml/min flow rate. The αA-WT protein partitioned into the soluble fraction was initially purified on a Hiload 16/60 superdex 200 gel filtration column equilibrated with 0.05 M PO_4_ buffer containing 0.15 M NaCl (pH 7.4). The αA-WT peak was pooled, concentrated, treated with solid urea (6M), and purified using an anion exchange column as described for the mutant protein. The purity of the proteins was checked by SDS-PAGE, and the molecular mass was determined by mass spectrometry. The concentration of the mutant and wild-type proteins was estimated using Bio-Rad protein assay reagent.

### 2.2 Isolation of βB_2_ Crystallin From β_L_-Crystallin Fractions

Frozen calf lenses were thawed, decapsulated, and added to 5.0 ml of 20 mM tris buffer, pH 7.9. The mixture was gently stirred for 30 min at 4°C and was then centrifuged at 12,000 rpm for 30 min. The supernatant was loaded onto a Sephadex G200 column to separate the β_L_-crystallin. The βB_2_-crystallin was isolated from the β_L_ fraction as described previously (Slingsby and Bateman, 1990). Briefly, the β_L_ fraction was concentrated to 5 mg/ml and equilibrated in tris buffer (pH 7.4) containing 6M urea and 2 mM tris(2-carboxyethyl) phosphine hydrochloride (Buffer A). The protein was then applied to Q Sepharose FF anion exchange column (GE Biosciences) equilibrated with buffer A, and the proteins were eluted using a linear salt gradient (0%–15%) of buffer A containing 1 M NaCl. The tubes containing the βB_2_—crystallin were pooled and concentrated. A small fraction of the protein was digested with trypsin, and the peptides were subjected to qTOF MS/MS analysis to confirm the protein.

### 2.3 Light Scattering Studies

Protein samples from various incubations (see fig legend) were injected (maximum volume 200 μl) onto a TSKgel G5000PW_XL_ (Tosoh Bioscience) size-exclusion column equilibrated with 0.05 M sodium phosphate buffer containing 0.15 M NaCl (pH 7.4). The flow rate was set to 0.75 ml. The column was attached to an HPLC system connected with UV and refractive index (RI) detectors and coupled to a static multi-angle laser light scattering (DAWN-EOS) and dynamic quasi-elastic light scattering (QELS) detectors (Wyatt Technology, Santa Barbara, CA, United States). The molar mass (M_w_), and hydrodynamic radius (R_h_), were determined using ASTRA (6.1) software developed by Wyatt Technology (Santa Barbara, CA, United States).

### 2.4 Chaperone Activity Measurements

The chaperone activity of αA-WT and αAG98R was compared using insulin, alcohol dehydrogenase (ADH), citrate synthase (CS) and βΒ_2_-crystallin (βΒ_2_) as substrates. The extent of aggregation was measured by monitoring the light scattering at 360 nm on a Shimadzu UV-VIS spectrophotometer equipped with a thermal controller. All assays were done in the absence or presence of different amounts of wild-type and mutant proteins in a 1 ml buffer.

#### 2.4.1 Insulin Aggregation

The insulin (0.15 mg; Sigma, St. Louis, MO, United States) aggregation was performed in 10 mM phosphate buffer, pH 7.4, containing 100 mM NaCl. Aggregation was initiated by adding 20 µl of 1 M DTT at 37°C.

#### 2.4.2 ADH Aggregation

ADH (250 μg; Worthington, Lakewood, NJ, United States) aggregation was induced by adding 100 mM EDTA in 0.05 M phosphate buffer containing 150 mM NaCl (pH 7.3) at 37°C.

#### 2.4.3 CS Aggregation

CS, 75 μg (Sigma, St. Louis, MO, United States) in 40 mM HEPES-KOH buffer (pH 7.4), was heated at 43°C. The aggregation of CS at 360 nm was measured up to 1 h.

#### 2.4.4 βB_2_-Crystallin Aggregation

The aggregation of βΒ_2_-crystallin (250 μg) in 0.05 M phosphate buffer containing 150 mM NaCl (pH 7.3) was measured at 37°C at various time intervals for up to 1 h.

### 2.5 Restriction Enzyme Stabilization Assay

The ability of αA-WT or αAG98R to stabilize SmaI restriction enzyme activity (New England Biolabs, USA) was tested as described by us earlier (Santhoshkumar and Sharma, 2001). *Sma*I (1 unit) was incubated in the presence or absence of 0.2 μg of αA-WT or αAG98R proteins at 37°C for 90 min. The incubation volume was adjusted to 10 μl using the buffer supplied by the enzyme manufacturer. After thermal inactivation, 200 ng of a plasmid containing three SmaI cleavable sites was added to the tube and incubated for 90 min at 25°C. The digested mixture was analyzed on 1% agarose gel.

### 2.6 Analysis of αA-WT and αAG98R Interactions With Substrate Proteins

The chaperone proteins were incubated with substrate proteins in aggregation assay buffers (250 μl) as defined earlier. A separate tube was set for each time point of analysis. For ADH (100 µg) and CS (20 µg) incubations, equal amounts of the chaperone proteins were used [1:1 ratio (w/w)]. For βB_2_-crystallin (150 µg) incubations, 25 µg of the mutant protein or 50 µg of αA-WT was used. The concentrations of the proteins selected were empirically determined. The incubation mixtures were analyzed at different time points as specified under the results section.

### 2.7 Modification of αA-WT and αAG98R by MGO

Chaperone proteins (1 mg/ml) in PBS were incubated with or without purified MGO (50 µM) (In-kind gift from Dr. Nagaraj) at 37°C in the dark under sterile conditions. The chaperone activity of the MGO modified αAG98R (25 µg) was analyzed using ADH (described in detail under results). Small aliquots (5 µg) were drawn at regular intervals and analyzed by SDS-PAGE analysis to check for crosslinking or autolysis. Another aliquot (25 µg) of the sample was analyzed by multi-angle light scattering, as described previously, to look for changes in the oligomeric mass distribution.

## 3 Results

### 3.1 αAG98R is Transiently Stabilized Upon Interaction With Substrate Proteins

**FIGURE 1:**
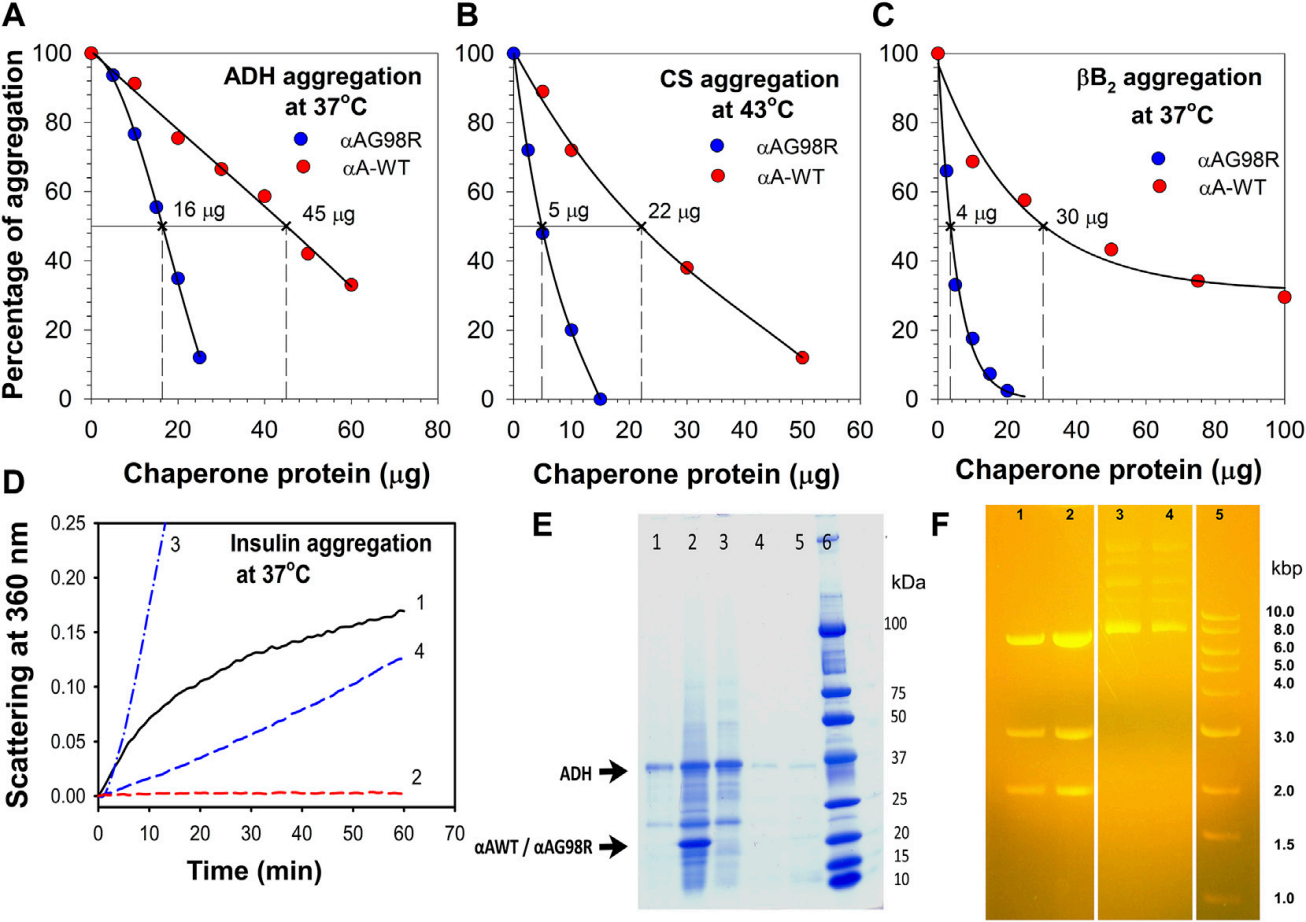
Relative functional activity of αAG98R and αA-WT proteins. The chaperone activity of αAG98R and αA-WT protein was compared at 60 min assay point and tested against—**(A)**, EDTA-induced aggregation of ADH at 37°C; **(B)**, thermal-induced aggregation of CS at 43°C; **(C)**, βB_2_-crystallin aggregation at 37°C. The aggregation of substrate protein shown at each concentration of chaperone protein tested is relative to the aggregation of substrate protein without the chaperone protein. The substrate protein aggregation (scattering at 360 nm) in the absence of chaperone protein is considered 100% aggregation. The EC_50_ values were calculated from the non-linear regression analysis obtained by plotting the % of substrate protein aggregation for a known chaperone protein concentration. Sigma plot (Version 12.5) (Systat Software Inc., Palo Alto, CA, United States) dynamic curve fitting with four-parameter logistic curve function was used for estimating EC_50_ values. The result shown is representative of three independent experiments. **(D)**, Relative chaperone activity of αAG98R and αA-WT towards DTT-induced insulin aggregation at 37°C. Curve1—Insulin (0.15 mg); Curve 2—Insulin (0.15 mg) + αA-WT (0.15 mg); Curve 3—Insulin (0.15 mg) + αA-G98R (0.15 mg); Curve 4—Insulin (0.15 mg) + αA-G98R (0.30 mg). **(E)**, SDS-PAGE analysis of chaperone assay samples left overnight at room temperature. ADH (0.1 mg) aggregation was performed in 1.0 ml buffer as described under methods and in the presence of 0.050 mg of αAG98R or αA-WT protein for 1 h (to achieve 100% suppression of ADH aggregation). The assay tubes left overnight at room temperature were centrifuged at 1,000 x g for 10 min to collect the pellet. 15.0 µl of the supernatant (without adjusting the sample volume after removing the sediments) and the entire pellet after solubilizing in 30 µl of loading dye were analyzed by SDS PAGE. Lane 1—[ADH + αA-G98R] supernatant; Lane 2—[ADH + αAG98R] pellet; lane 3—ADH—2.5 µg; Lane 4—[ADH + αA-WT] pellet; Lane 5—[ADH + αA-WT] Supernatant. The small impurities present in the ADH (lane 3) are unlikely to change the observed results. The strong band for ADH in lane 1 suggests that a significant amount of the protein remained soluble without interacting with the chaperone protein. **(F)** αAG98R protects SmaI from thermal inactivation. Agarose gel electrophoresis showing the DNA digestion profile of SmaI heated at 37°C for 90 min in the presence or absence of chaperone proteins. Lane 1—[SmaI (1 unit) + αA-WT (0.2 µg)]; Lane 2—[SmaI (1 unit) + αAG98R (0.2 µg)]; Lanes 3 & 4—SmaI (1 unit); Lane 5—marker. Three bands in lanes 1 & 2 indicate that the SmaI is active and the digestion is complete.

Previously, studies have shown that αAG98R is prone to aggregate rapidly at near-physiological temperatures. Comparing the chaperone activity of αA-WT and the mutant protein we reported earlier that the mutant protein shows a substrate-dependent chaperone activity (Murugesan et al., 2007; Raju et al., 2011). This study involved multiple concentrations of the αA-WT and mutant protein and different client proteins and assay conditions to estimate relative chaperone efficiency (EC_50_) of both chaperone proteins. In thermal aggregation assays, where the aggregation of substrate proteins occurred slowly, the mutant was more efficient than the αA-WT in preventing the aggregation of substrate proteins (Figure 1). Based on the EC_50_ values determined by comparing the scattering at 60 min assay time point, αAG98R (EC_50_—16 µg) (Figure 1A) was nearly three times more efficient than the WT protein (EC_50_—45 µg) in preventing ADH aggregation. For CS, fifty percent protection from aggregation was obtained with 5 µg of the mutant protein (Figure 1B). The mutant was over four times better in protecting CS aggregation when compared to the αA-WT which, exhibits similar protection of CS at 22 µg. Similarly, 30 µg of αA-WT was required to suppress the aggregation of βB_2_ by 50%, whereas 4 µg of the mutant protein was able to provide the same level of protection (Figure 1C). However, in assays where the substrate protein aggregates rapidly, like the DTT-induced insulin aggregation, αA-WT showed better chaperone activity than the mutant protein (Figure 1D). While the DTT-induced insulin aggregation was entirely prevented by αA-WT when used at a 1:1 (w:w) ratio (Figure 1D, Curve 2), the G98R mutant protein at the same amount could not provide any protection against insulin. Instead, it increased the light scattering by insulin (Figure 1D, Curve 3), and when doubling the amount of mutant protein, a 25% suppression of insulin aggregation was seen (Figure 1D, Curve 4). Although αAG98R was better than the WT protein in suppressing ADH aggregation during the assay period (Figure 1A), the substrate protein complexes formed with the mutant (in tubes showing 100% protection during chaperone assay) precipitated when the chaperone assay samples were left overnight (16 h) at room temperature (Figure 1E, Lane 2). The tubes with αA-WT showing a similar level of protection did not show any visible deposits when left overnight (16 h) at room temperature (Figure 1E, Lane 4). Next, we tested if αAG98R can protect from the thermal inactivation of restriction endonuclease, SmaI, at 37°C. The mutant protein completely protected the SmaI from heat inactivation (Figure 1F, Lane 2), just like the αA-WT (Figure 1F, Lane 1). In the absence of chaperone protein, SmaI incubated at 37°C failed to cleave the DNA (Lanes 3 & 4). Our data confirm the substrate-dependent chaperone activity of αAG98R mutant. The results also suggest that αAG98R protein, which is unstable at near-physiological temperatures, gets transiently stabilized upon interaction with specific substrate proteins.

### 3.2 αA-WT and αAG98R Mutant Proteins Interact Differently With the Substrate Proteins

We followed the interactions of αA-WT and the mutant with the substrate proteins (ADH, CS, and βB_2_) on a MALS detector at regular intervals to analyze the complex formation during chaperone action at 37°C. Excess of chaperone proteins was used to ensure that the substrates did not scatter light (A360 nm) during the duration of the experiment. The incubations were carried out in appropriate aggregation buffers (0.25 ml) and in separate tubes for each time point of analysis. All samples were filtered through 0.45 µm Whatman® Mini-UniPrep® syringeless filters (Sigma, St Louis, MO, United States) before fractionating on a TSKgel G5000PW_XL_ column connected to an HPLC coupled with MALS-DLS detectors. Under the defined incubation conditions, CS aggregated slowly, so we decided to follow the complex formation for up to 20 h. ADH and βB_2_ samples were followed up to 90 min. ADH and βB_2_ showed a scatter of (Absorbance 360 nm) about 0.35 and 0.06, respectively, in the absence of chaperones at 90 min.

**FIGURE 2:**
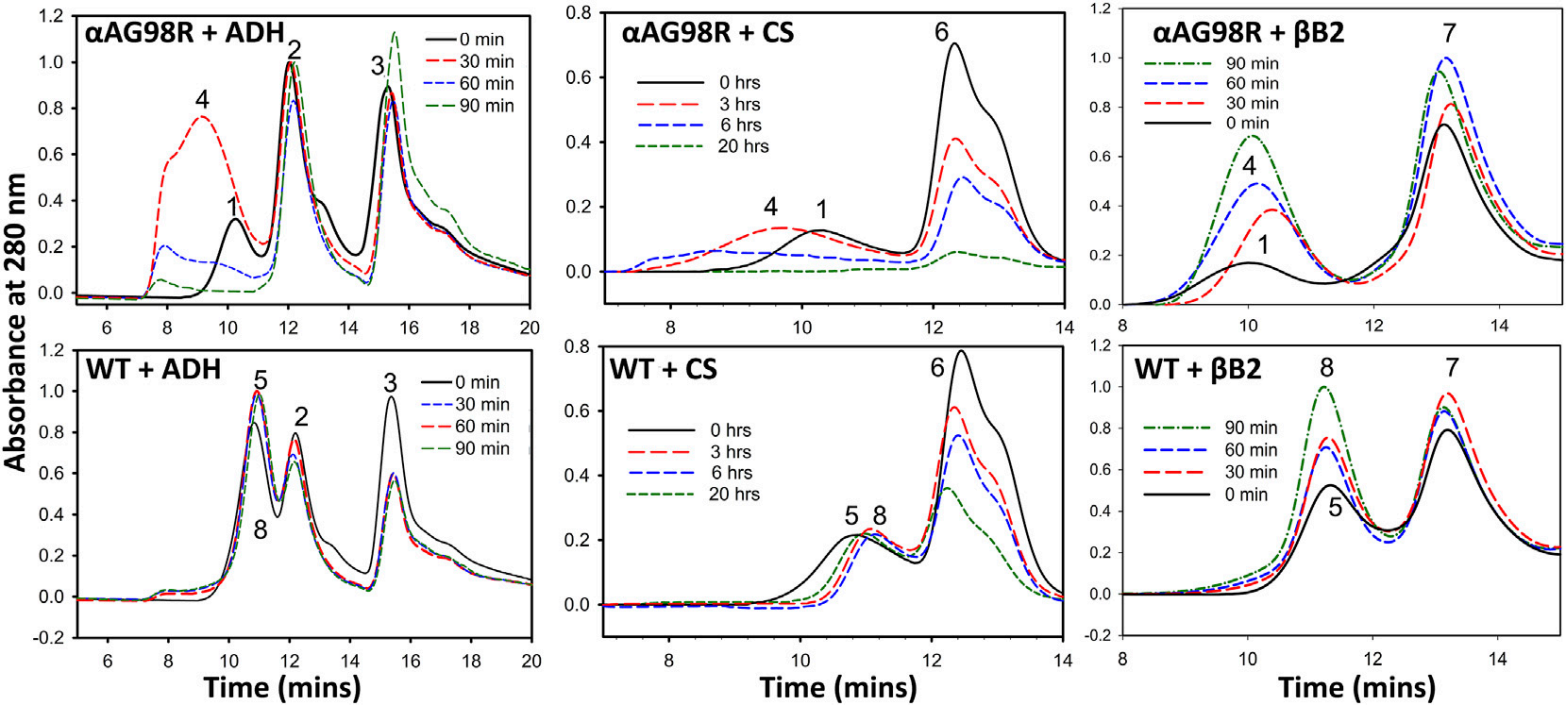
Gel filtration elution profiles of incubation mixtures of substrate proteins with αAG98R or αA-WT at different time intervals. The peaks are identified as follows 1—αAG98R or complex at 0 min; 2—ADH tetramer; 3—ADH monomer; 4—αAG98R-substrate complexes; 5—αA-WT or complex at 0 min; 6—CS; 7—βB_2_-crystallin; 8—αA-WT-substrate complexes. Only the area under peaks 1, 4, 5, and 8 are analyzed using ASTRA software, and the results are summarized in Table 1.

Figure 2 shows the elution profiles of the samples from the UV detector. The average molar mass (M_w_) and the hydrodynamic radii (R_h_) at the complex peak determined from the light scattering detectors using the ASTRA software are given in Table 1. A slight shift in the αA-WT peak with ADH was seen in samples analyzed at 90 min (Figure 2, Peak 8) when compared to the peak at 0 min (Figure 2, Peak 5), which correlated with a corresponding increase in M_w_ from 824 kDa (0 min) to 1,192 kDa (90 min) and a rise in R_h_ from 7.8 to 12.8 nm (Table 1). We also found some high molecular weight complexes eluting at 7–9 min. However, the protein concentration was too low to analyze the M_w_. In contrast to the WT, the mutant protein formed large oligomers with ADH, and the complex size (R_h_) increased with time from 12.5 nm at 0 min to 38 nm at 90 min. We could only determine the Mw at 0 min, after which the light scattering signal maxed out, hampering M_w_ analysis.

**TABLE 1:** Multi-angle light scattering analysis of chaperone and substrate protein incubation mixtures carried out at different time points.

	ADH				CS				βB2			
	Incubation time	Peak	Molar mass	Hydrodynamic Radii	Incubation time	Peak	Molar mass	Hydrodynamic Radii	Incubation time	Peak	Molar mass	Hydrodynamic Radii
	Min	Min	Mw (kDa)	Rh (nm)	Hrs	Min	Mw (kDa)	Rh (nm)	Min	Min	Mw (kDa)	Rh (nm)
αA-WT	0.00	10.50	824 ± 01	7.81 ± 0.19	0.00	10.71	822 ± 05	8.45 ± 0.42	0.00	11.21	642 ± 09	7.35 ± 0.21
	30.00	10.78	1,113 ± 09	10.17 ± 0.23	3.50	11.04	589 ± 07	NR	30.00	11.24	683 ± 11	7.32 ± 0.20
	60.00	10.90	1,171 ± 09	11.93 ± 0.29	7.00	11.04	378 ± 08	NR	60.00	11.17	688 ± 10	7.69 ± 0.20
	90.00	10.91	1,192 ± 11	12.76 ± 0.33	20.00	10.91	851 ± 13	NR	90.00	11.18	822 ± 08	7.70 ± 0.2
αAG98R	0.00	10.27	2,612 ± 06	12.53 ± 0.31	0.00	10.17	2,452 ± 12	13.49 ± 0.50	0.00	9.72	9,520 ± 10	17.83 ± 0.43
	30.00	9.25	NR	25.57 ± 0.63	3.50	9.54	12,470 ± 20	25.22 ± 0.59	30.00	9.94	5,460 ± 05	14.23 ± 0.33
	60.00	8.01	NR	30.76 ± 0.82	7.00	8.54	161,900 ± 110	35.09 ± 0.80	60.00	9.92	5,472 ± 04	14.77 ± 0.35
	90.00	7.83	NR	38.25 ± 0.85	20.00	NR	NR	NR	90.00	9.97	4,484 ± 04	13.47 ± 0.33

The data acquired from RI and DAWN-QELS detectors were analyzed using ASTRA (6.1) software developed by Wyatt Technology. The UV elution profiles of the samples are shown in Figure 2. “NR” indicates data is unreliable. The protein concentrations were in nanogram levels, or the light scattering detector signal maxed out, preventing accurate mass measurements. Only the complex peaks identified in Figure 2 were analyzed.

With CS, αAG98R showed a similar effect, and the R_h_ increased from 13 to 35 nm, and M_w_ increased from 2,452 kDa to 161,900 kDa in 0–7 h (Table 1). The sample at 20 h could not be analyzed due to high scattering and low protein concentrations. Most of the aggregates were likely removed during the initial filtering of the sample. In contrast to αAG98R, the interaction with CS did not increase the oligomeric mass of αA-WT. Instead, the M_w_ at the peak apex reduced from 822 to 589 kDa in 3.5 h, and it decreased further to 378 kDa in 7 h (Table 1). However, further incubation of the sample up to 20 h showed the complex peak mass increasing to 851 kDa. The reduction in the CS + αAWT complex peak mass is supported by delay in the elution of complex peak (Peak 8, Figure 2 lower center) with time. Since the data points from the DLS detector did not fit well, the R_h_ values reported by the ASTRA software for these samples were not considered.

The UV profile of αAG98R and βB_2_ at 0 min showed the mutant peak (Figure 2, Peak 1, the upper panel right) appearing early at 9.72 min, unlike in ADH and CS incubations, the mutant was seen eluting at 10.2 min (Table 1). The early appearance of the mutant protein in the βB_2_ sample is also supported by an increase in M_w_ (9,520 kDa) and R_h_ (17.83 nm) (Table 1) at the peak apex, suggesting that αAG98R and βB2-crystallin interact during the duration (<5 min) of sample preparation. Surprisingly, continuing the incubations further did not result in high molecular weight species as seen with ADH and CS. Instead, there was a significant decrease in M_w_ and R_h_ of the complex peak (Table 1). The αAG98R-βB_2_ peak intensity also progressively increased with time, unlike the peak intensities of complexes with the other two substrates (Figure 2, upper panel). The interaction of αA-WT with βB_2_ did not significantly alter the M_w_ and R_h_ of the complex peak up to 60 min. However, at 90 min, the M_w_ slightly increased from 688 to 822 kDa (Table 1).

### 3.3 αAG98R Interactions Alter the Oligomerization of Lens Proteins

**FIGURE 3:**
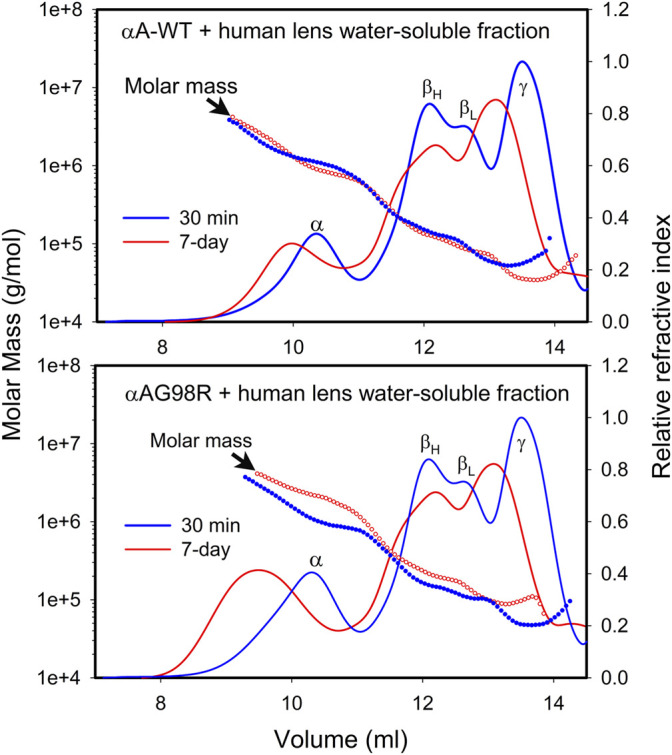
Mass distribution in 16-year old human lens water-soluble extract incubated with αAG98R or αA-WT. The water-soluble lens extract containing 250 µg protein was incubated with 50 µg of the chaperone protein under aseptic conditions in 0.25 ml PBS. At the end of the incubation, the sample was filtered to remove any residues, and 150 µl was injected into a TSKgel G5000PW_XL_ size exclusion column attached to a Shimadzu HPLC system with a RI detector. The mobile phase contained 0.05 M sodium phosphate and 0.15 M sodium chloride, pH 7.2. The eluting proteins were analyzed with the help of WYATT MALS-DLS detectors.

Using multi-angle light scattering, we have investigated whether incubating αAG98R with the water-soluble fraction of young human lenses (donor lenses obtained from the Lions Eye Tissue Bank, Columbia, MO) can alter the mass distribution during sample aging. The water-soluble fraction from a 16-year-old human lens (250 µg protein) (Santhoshkumar et al., 2008) was incubated under aseptic conditions with 50 µg of αA-WT or mutant protein in 0.25 ml PBS containing 0.03% sodium azide and protease inhibitors (Sigma, St. Louis, MO, United States) at 37°C for 7 days. At the end of the incubation, the sample (150 µl) was processed for MALS analysis as described previously. Another set of samples was prepared and analyzed after 30 min incubation at 37°C. The mass distribution across the refractive index (RI) profiles of the incubation mixtures at 30 min and 7 days was compared (Figure 3). As expected, the lens proteins treated with αA-WT or mutant protein for 7 days (red curve) eluted early from the column compared to the freshly analyzed (30 min) sample (blue curve), suggesting the formation of high molecular weight aggregates during sample aging *in vitro*. In sample(s) with αA-WT, the mass distribution across the RI profile of both time points was more or less overlapping, indicating no significant change in the mass of the complexes during the incubation period. The elution profile and mass distribution in incubations of lens extract alone (data now shown) were like in lens extract incubated with αA-WT. In contrast, samples incubated with αAG98R showed a differential mass distribution with the 7-day incubation mixture showing higher molar mass across the RI profile (Figure 3 bottom). The aged sample with αAG98R also had large oligomers eluting in 7–9 ml. This caused the light scattering detector to max out so we could not show the mass distribution in that region. Overall, the magnitude of aggregates was increased when αAG98R was added to the lens extract. The aggregates’ size was comparable to those found in water-soluble aged lens extracts (Santhoshkumar et al., 2008), implying that G98R mutation in αA could accelerate lens aging.

### 3.4 Incubation With Low Concentrations of MGO Stabilizes αAG98R Protein

**FIGURE 4:**
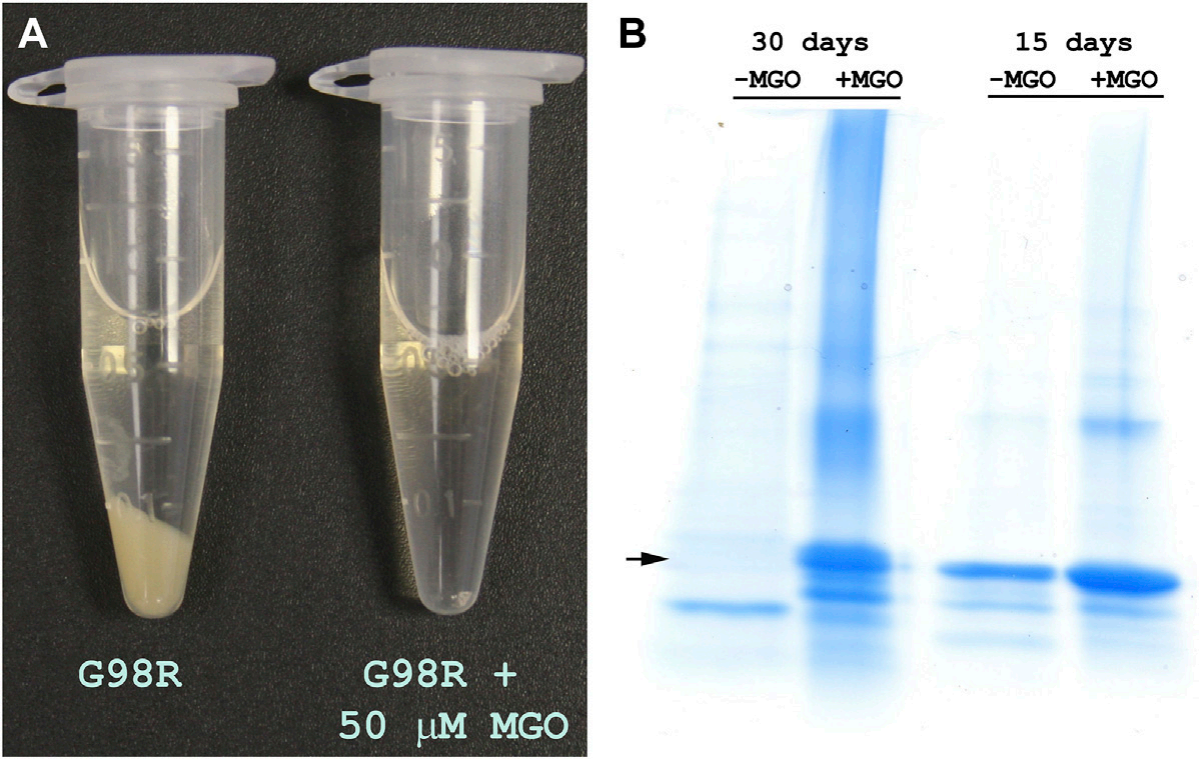
Modification by MGO improves the stability of αAG98R protein. **(A)** Image of samples incubated for 30 days showing the precipitation of the mutant protein in the absence of MGO and in the presence of 50 µM MGO, the sample remained clear. **(B)** SDS-PAGE analysis of incubation mixture of αAG98R in the presence or absence of MGO. In the sample without MGO, there was a complete breakdown of the mutant protein in 30 days. A strong band for the intact protein was seen in the sample with MGO. A small amount of crosslinked and cleaved protein bands were also seen in this sample.

Previous studies have shown that MGO modification of arginine residues neutralizes the positive charge (Derham and Harding, 2002; Biswas et al., 2008). Since G98R mutation results in the gain of positive charge, we investigated whether incubation of the mutant protein with low MGO concentrations would improve the mutant protein’s stability. The αAG98R protein (1 mg/ml) was incubated at 37°C under aseptic condition (filtering through a 2 µm filter) without or with 50 µM MGO in PBS containing 0.03% sodium azide for 30 days. The samples were visually observed for sediments and were analyzed by SDS-PAGE every week to check for breakdown/crosslinking. In the tube without MGO, a large deposit was seen at the bottom at 30 days, while the sample with MGO remained transparent (Figure 4A). SDS-PAGE analysis showed that the mutant protein shows autolytic activity without MGO (Figure 4B). More than 50% of the mutant protein band was reduced in 15 days without MGO, and at 30 days, the band for full-length αAG98R protein was completely absent and a prominent band for a cleavage product was seen. In contrast, the mutant protein incubated with MGO did not show any breakdown at 15 days, but a small amount (<5%) of crosslinked proteins were seen. In the 30-day sample, the crosslinks increased slightly, and some faint bands below the prominent intact protein band were seen. In general, the data suggest that low concentrations of MGO can stabilize αAG98R mutant protein.

### 3.5 Methylglyoxal Modifications of αAG98R Reduces the Aggregation Propensity of the Mutant Protein

**FIGURE 5:**
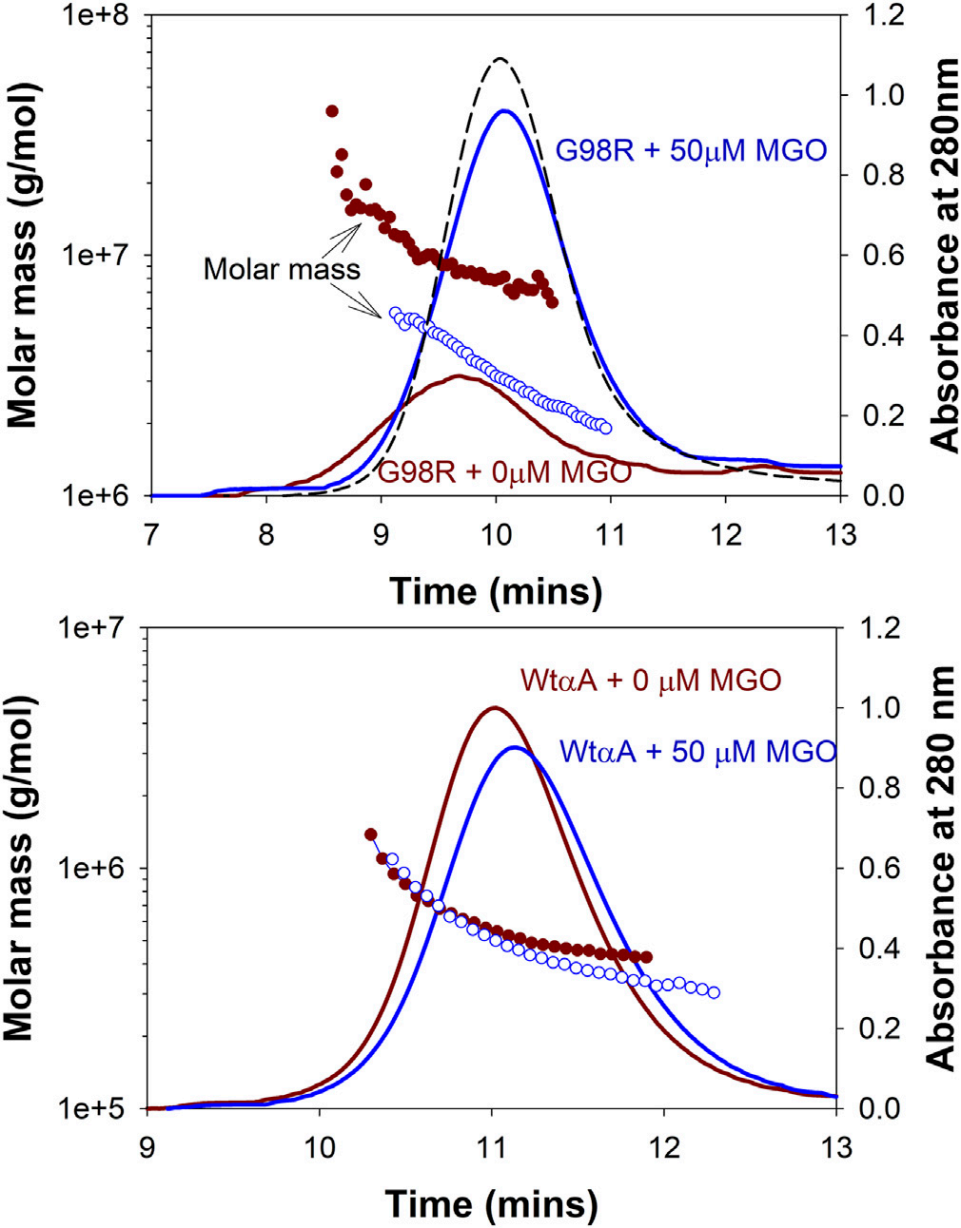
Effect of MGO modifications on the molar mass distribution of αAG98R and αA-WT proteins. The proteins were treated with 50 µM MGO for 7 days. MGO treatment reduces the aggregation propensity of αAG98R protein but does not significantly affect αA-WT oligomers. The results of light scattering analysis are summarized in Table 2. The black (curve dashed) is the elution profile of untreated αAG98R at 0 min. The protein concentration of this peak reported from the RI detector is considered 100% recovery for mutant protein. The αA-WT peak at 0 min overlapped with the (αA-WT + 0 mM MGO) peak and is not shown.

We next investigated the effect of MGO modifications on the molar mass distribution of αAG98R by MALS analysis. Figure 5 shows the mass distribution across the UV profiles of αA-WT and αAG98R proteins incubated with or without 50 µM MGO at 37°C for 7 days. The M_w_ and R_h_ calculated from the MALS-DLS signals using the ASTRA software are given in Table 2. In the absence of MGO, αAG98R mainly precipitated and was removed during sample filtering. Only 18% of the protein remained soluble and eluted as a broad peak (Figure 5 upper panel–red) consisting of G98R oligomers with mass ranging from 41,302–5,730 kDa (Table 2). In contrast, the mutant protein incubated with MGO showed a prominent peak (Figure 5 upper panel—blue), with 80% of the protein recovered in the soluble form (compared to the sample analyzed at 0 min). The average molar mass of the soluble αAG98R oligomers decreased from 9,386 kDa to 3,350 kDa in the presence of MGO at 7 days (Table 2). The hydrodynamic radii also showed a similar decrease in size (Table 2). The elution profile of αAG98R without MGO at 0 min (shown in black dash line for comparison) had an average oligomer mass of 2057 kDa (data not shown). Unlike as seen with αAG98R, MGO modifications of αA-WT did not show a significant reduction in M_w_ and R_h_. The average M_w_ of αA-WT decreased by about 100 kDa, and the size was reduced by 0.1 nm in samples with MGO. The data suggest that MGO modifications reduce the aggregation propensity and improve the solubility of αAG98R mutant protein. The residues that MGO modifies in αAG98R are yet to be investigated.

**TABLE 2:** Multi-angle light scattering analysis of αAG98R protein treated with MGO for 7 days.

Incubations	Molar mass range M_w_ (kDa)	Average mass M_w_ (kDa)	Average R_h_ (nm)	Soluble protein recovered (%)
αAG98R + 0 µM MGO—7 days	41,302–5,730	9,386 ± 18	21.7 ± 0.8	18
αAG98R + 50 µM MGO—7 days	6,726–1950	3,350 ± 09	13.1 ± 0.3	80
αAWT +0 µM MGO—7 days	1,340–432	562 ± 06	7.6 ± 0.1	76
αAWT +50 µM MGO—7 days	1,030–318	467 ± 05	7.5 ± 0.1	80

The elution profiles of the samples used for analysis are shown in Figure 5. The data acquired from RI and DAWN-QELS detectors were analyzed using ASTRA (6.1) software developed by Wyatt Technology. The protein concentration was determined from RI peak signals. The protein concentration of samples determined at 0 min was considered 100% recovery.

### 3.6 Methylglyoxal Modified αAG98R Shows Improved Anti-Aggregation Activity

**FIGURE 6:**
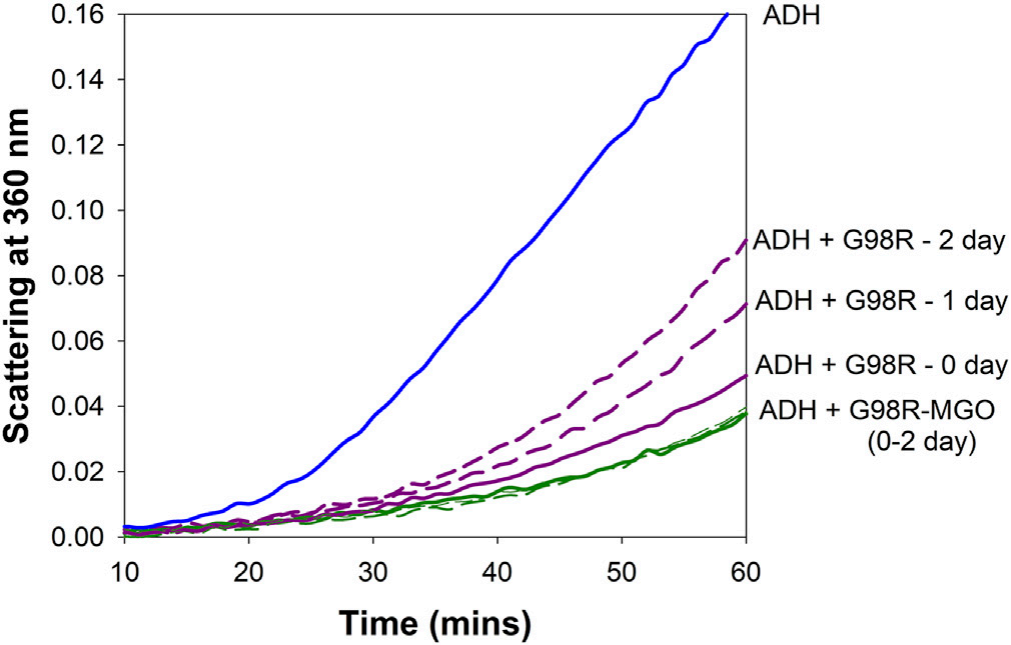
MGO modification improves the chaperone activity of αAG98R protein. The incubations were set up at different periods to carry out the chaperone assay of all the samples simultaneously. In the absence of MGO, the ability of αAG98R (25 µg), incubated at 37°C, to suppress EDTA-induced aggregation of ADH (250 µg) progressively decreases with the incubation duration. At the same time, αAG98R modified by 50 µM MGO showed improved chaperone activity and was maintained during incubation.

Previous studies have shown that incubations with low concentrations of MGO will improve the chaperone function of α-crystallin (Nagaraj et al., 2003; Nagaraj et al., 2008). We have tested if αAG98R modification with MGO will alter the anti-aggregation property of the mutant protein. The mutant protein (1 mg/ml) was incubated with or without 50 µM MGO as described previously. The chaperone activity of the samples was measured immediately (0-time point) and at one and 2 days after the start of the incubation using ADH (250 µg) as the aggregating client protein. Before the chaperone assay, the free MGO in the sample was removed by quick buffer exchange (3x) using a 10 kDa Vivaspin 2 ultra-concentrator (Sartorius, Bohemia, NY, Unites States). Samples without MGO were also processed similarly. The protein concentrations were estimated using Bio-Rad protein assay. The anti-aggregation activity was tested using 25 µg of the mutant protein. The chaperone activity of unmodified αAG98R reduced with time, while that of the MGO-modified mutant protein remained the same at all time points (Figure 6). αAG98R treatment with MGO also slightly increased the anti-aggregation activity compared to the untreated protein. Our data suggest that the chaperone activity of αAG98R is improved by modification with MGO.

## 4 Discussion

Arginine residues play an essential role in the structure and function of α-crystallin. Modifications of specific arginine residues in crystallins cause pathological conditions. Most human congenital cataracts are caused by the mutation of arginine residues (loss of a positive charge) in αA-crystallin (Raju et al., 2011; Panda et al., 2016). The G98R mutation is the only reported “gain of charge” mutation in human αA-crystallin linked to a pathological condition. Unlike congenital cataracts seen in the “loss of charge” mutants, the onset of cataracts occurs in the late teens of individuals carrying the αAG98R mutation (Santhiya et al., 2006). An earlier study has shown that G98R mutation in αA-crystallin results in an altered structure that tends to aggregate (Singh et al., 2006). Using a single concentration of the chaperone protein and testing carried out only with insulin substrate, they reported that αAG98R lacks the chaperone function. During the same period, we have compared the anti-aggregation activity of the αAG98R with αAWT using multiple substrates and reported that αAG98R mutant exhibits substrate-dependent chaperone activity (Murugesan et al., 2007). The present study compared the effectiveness of αAWT and αAG98R proteins in suppressing the aggregation of substrate proteins unfolded by different methods and analyzed the complexes formed during the chaperone action. The lower EC_50_ value of the mutant protein suggests that αAG98R is more efficient than αAWT in suppressing the aggregation of ADH, CS, and βB_2_-crystallin (Figures 1A–C). As reported in the earlier study (Singh et al., 2006), αAG98R showed an increased scattering of insulin when used at a 1:1 ratio (w/w). Doubling the mutant protein concentration suppressed the aggregation of insulin by 25%. We observed that G98R protected SmaI restriction endonuclease from thermal inactivation like the WT protein. The results support our earlier study, which reported αAG98R protein has a substrate-dependent chaperone activity (Murugesan et al., 2007). However, the protection offered by the αAG98R appeared to be temporary as the complexes of G98R with ADH in chaperone assays precipitated when left overnight at room temperature (Figure 1E). The data confirm that cataract development in individuals with αAG98R mutation is not due to the loss of chaperone function, but perhaps due to gain of function and aggregation of the chaperone-substrate complexes into light scattering particles. The study also suggests that an empirical evaluation of the chaperone activity would require testing using several concentrations of the chaperone protein and with multiple substrates. The improved chaperone activity of the mutant protein could be attributed to its enhanced affinity towards the substrate protein. The mutant protein likely interacts with substrates having partially non-native states resulting in increased association (Koteiche and McHaourab, 2006). The increase in the M_w_ of the freshly prepared αAG98R-βB_2_ complex (peak at 0 min, Table 1) supports this view.

The αAG98R has an unstable structure at physiological temperatures, and the oligomers form larger aggregates with time and eventually precipitate (Murugesan et al., 2007; Raju et al., 2011). The aggregation propensity of the αAG98R protein is transiently alleviated in the presence of substrate proteins. This view is supported by absence of the visible precipitates in the chaperone assay tubes that contained αAG98R + ADH complexes. Incubation of αAG98R alone under similar conditions results in the mutant protein precipitation (Murugesan et al., 2007; Raju et al., 2011). The stabilization of mutant protein is also seen when incubated with water-soluble lens proteins (Figure 3), likely due to the interaction with other lens proteins. Furthermore, the autolysis of αAG98R seen during prolonged incubation (Figure 4) was not seen in the presence of lens proteins (data not shown), and the sample was clear. An earlier study has established that mixed oligomers of αA-WT and αAG98R have a decreased propensity to aggregate compared to the homo-oligomers of the mutant protein, and the properties of the mixed oligomer are dominated by those of the mutant protein (Singh et al., 2007). In the present study, the mass distribution of mixtures of lens extract with G98R incubated for 7 days showed higher mass proteins across the RI profile (Figure 3, bottom), suggesting that the mutant protein interacts with multiple lens proteins.

MALS analysis of the chaperone-substrate protein incubation mixtures revealed that αA-WT protein forms more compact complexes while chaperoning the misfolded proteins resisting a significant increase in the complex size (Table 1). The αA-WT likely maintains the complex size by reorganizing monomers and exchanging the subunits with the substrate protein (Bova et al., 1997). The G98R mutation affects the ability of αA-crystallin to maintain the oligomeric size resulting in high molecular weight oligomers formed by themselves or upon interacting with misfolded proteins. It appears that mutant subunits are loosely held in the oligomers resulting in less compact complexes with the substrates (Figure 2). Our results suggest that the binding of αAG98R to the substrate briefly stabilizes both proteins, but the complexes retain the aggregation property of the mutant protein. The size of the complexes formed with the mutant seems to depend on the aggregation kinetics of the substrate proteins. A substrate showing increased light scattering (ADH) formed large complexes with the mutant protein than a substrate that aggregated slowly (βB_2_). Surprisingly in the incubations of mutant protein with βB_2_, the oligomeric size of the complex peak was seen decreasing with time (Table 1). The βB_2_ that we isolated from the lens might have a partial non-native structure prompting a rapid interaction of the mutant protein. We propose that as βB_2_ unfolds, the G98R subunits likely reorganize, resulting in a smaller oligomeric size. Further experiments are needed to confirm this and to see if, on prolonged incubation, the complex will adopt the mutant proteins aggregation property as in the case of ADH and mutant complex.

Previous studies have suggested that low concentrations of MGO might benefit lenses and can be harmful at high concentrations (Nagaraj et al., 2003; Satish Kumar et al., 2004). MGO at low concentrations primarily targets arginine residues in αA-crystallin, causing an overall loss of positive charge (Gangadhariah et al., 2010). Partial modification of αA-crystallin by MGO enhances the chaperone function (Nagaraj et al., 2003; Gangadhariah et al., 2010). The present study has shown that MGO modifications of G98R improve the protein’s stability and reduce the autolytic activity (Figure 4). MGO modified G98R protein showed decreased aggregation propensity and improved solubility (Figure 5; Table 2). The chaperone activity of the αAG98R is preserved when modified with MGO (Figure 6). A previous study has shown that at 20 µM MGO, 4 arginine residues (R12, R65, R157, and R163) are modified, and at 500 µM MGO, 11 arginine residues in αA-crystallin are modified to hydroimidazolone (Gangadhariah et al., 2010). We have yet to identify the residues targeted by MGO in the αAG98R mutant. Even if we do not see the modification of R98 residue with 50 µM MGO, changes caused by the alteration of other arginine residues in αAG98R by MGO might be sufficient to rescue from the effects of the mutation, as seen in this study. Recently we showed that a targeted compensatory suppressor mutation (R21Q) in the N-terminal can salvage from the harmful impacts of αAG98R (Phadte et al., 2019). Although MGO modification reduced the oligomeric size of αAG98R (21.7–13.1 nm), it was significantly higher than MGO modified αA-WT protein (7.6 nm). Our results suggest that MGO modification cannot prevent cataract formation but could delay its appearance at birth. Thus, the beneficial effects of MGO seen *in vitro* using the recombinant mutant protein may not be translated fully to *in vivo* conditions to provide life-long protection from cataractogenesis.

In conclusion, our study confirms the earlier observation that the cataract development in αAG98R mutant is due to the altered chaperone function of the mutant protein. The study further adds that the substrate protein’s aggregation kinetics, the affinity of the chaperone to a substrate protein, the extent of interactions, and the complex stability, all seem to contribute to the chaperone efficiency of αAG98R protein. The mutant protein is transiently stabilized, and the mutant’s aggregation propensity is reduced upon interation with substrate protein and MGO modification. These observations may provide a molecular basis for presenile cataract formation in individuals with αAG98R mutation.

## Data Availability

The raw data supporting the conclusion of this article will be made available by the authors, without undue reservation.
